# “Now, I have my baby so I don’t go anywhere”: A mixed method approach to the ‘everyday’ and young motherhood integrating qualitative interviews and passive digital data from mobile devices

**DOI:** 10.1371/journal.pone.0269443

**Published:** 2022-07-08

**Authors:** Ashley Hagaman, Damaris Lopez Mercado, Anubhuti Poudyal, Dörte Bemme, Clare Boone, Alastair van Heerden, Prabin Byanjankar, Sujen Man Maharjan, Ada Thapa, Brandon A. Kohrt

**Affiliations:** 1 Department of Social and Behavioral Sciences, Yale School of Public Health, Yale University, New Haven, Connecticut, United States of America; 2 Center for Methods in Implementation and Prevention Science, Yale School of Public Health, Yale University, New Haven, Connecticut, United States of America; 3 Division of Global Mental Health, Department of Psychiatry and Behavioral Sciences, George Washington School of Medicine and Health Sciences, Washington, DC, United States of America; 4 Department for Global Health and Social Medicine, Centre for Society & Mental Health, King’s College London, London, United Kingdom; 5 Yale University, New Haven, Connecticut, United States of America; 6 Human and Social Development, Human Sciences Research Council, Pietermaritzburg, South Africa; 7 Medical Research Council/Wits Developmental Pathways for Health Research Unit, Department of Pediatrics, Faculty of Health Sciences, University of the Witwatersrand, Johannesburg, South Africa; 8 Transcultural Psychosocial Organization (TPO) Nepal, Kathmandu, Nepal; 9 George Washington School of Medicine and Health Sciences, Washington, DC, United States of America; University of Huddersfield, UNITED KINGDOM

## Abstract

The impacts of early pregnancy and young motherhood on everyday life, including interpersonal and individual behavior, are not well-known. Passive digital sensing on mobile technology including smartphones and passive Bluetooth beacons can yield information such as geographic movement, physical activity, and mother-infant proximity to illuminate behavioral patterns of a mother’s everyday in Nepal. We contribute to mixed-methods research by triangulating passive sensing data (GPS, accelerometry, Bluetooth proximity) with multiple forms of qualitative data to characterize behavioral patterns and experiences of young motherhood in the first year postpartum. We triangulated this digital information in a constant comparative analysis with in-depth interviews, daily diaries, and fieldnotes. We reveal typical behavioral patterns of rural young mothers and highlight opportunities for integrating this information to improve health and well-being.

## Introduction

Low- and middle-income countries (LMIC) have high rates of adolescent marriage and pregnancy and persistent gender disparities in health and social outcomes, where women marry younger, initiate sexual activity younger, and are afforded less autonomy and agency in pursuing educational and economic opportunities, particularly when they enter early motherhood [1–3]. While motherhood is indeed a valued and important life transition for many, the perinatal period and added responsibility of childrearing heightens women’s vulnerability to a range of health conditions. Persistent poverty, discrimination and violence, suboptimal psychosocial health, and poor physical health compounds risk faced by young mothers and special attention to this high-risk group is especially critical [4, 5]. Previous research in young maternal populations has largely focused on health-specific indicators, however, a focus on the ‘every day’ (where a woman goes, who she interacts with, how she engages and cares for her baby) might illuminate important components of health and behavior and provide important intervention targets for a range of health conditions.

Nepal has the second highest rate of adolescent pregnancy in South Asia [6], with approximately 40 percent of women becoming mothers before the age of 20 [7]. Childbirth is an important life event for Nepali women, fulfilling a seminal social role and providing upward social mobility [8, 9]. Generally, Nepali culture follows a patrilocal tradition where, upon marriage, women leave their maternal household (*maiti*) and become permanent members of their husband’s parents’ household [10]. The woman’s husband and in-laws typically have prominent decision-making power related to her reproduction healthcare, and social engagement [11]. These stressors may be compounded for women that marry at a young age, with pervasive effects limiting her educational, socioeconomic, and empowerment trajectories.

Understanding the cultural and social dimensions of a mother’s typical life is crucial in determining the most effective ways to deliver appropriate health interventions to adolescent and young mothers. Recent advancements in digital technologies provide a new avenue to better understand young maternal behavior through passively collected digital behavioral data from a smartphone or wearable device. This real-time information can be a powerful tool for the management of chronic disease [12, 13]. Despite rapid advances in passive digital health research, little research has illuminated specific mechanisms in behavioral patterns and decisions, particularly among vulnerable, low-income populations. In perinatal women, passive monitoring movement patterns may be useful in identifying women at risk for depression [14, 15]. More research is needed to identify and validate associations between specific digital biomarkers and perinatal behaviors. Further opportunities that leverage mixed-methods to harness movement patterns of mothers as a tool for capturing their day-to-day experiences remain.

Social interactions are also a target of inquiry with passive sensing technologies, measured using audio recordings [16], phone usage data [17], and Bluetooth beacons [18, 19]. Social features derived from audio data are associated with measures of social connectedness, which is a promising indication of their validity [20]. Social environment has been examined as an important factor in overall well-being [20], and opportunities exist to examine what measures of social interactions with infants might tell us about the experiences of perinatal women. In postpartum women, proximity has been crudely measured by room sharing or if the mother lives with her infant or not [21, 22]. To date, studies have not systematically examined continuous infant proximity throughout the day nor how these patterns might be related to other aspects of maternal wellbeing.

In the existing literature of passive sensing technologies, most studies focus on identifying digital markers of specific disease states and symptoms, but passively collected sensing data also holds enormous potential to unobtrusively capture the daily lives of individuals. The wealth of data on an individual’s unique environment can help us understand lived experiences, identify needs, and tailor interventions. This study seeks to demonstrate the value of using mixed methods to construct a holistic view of women’s daily lives. In addition, there is a dearth of research focusing on women in LMIC—their digital phenotypes may be substantially different from those of women in HIC given the different daily tasks and social roles of women. This paper contributes to filling this gap to better understand the daily experiences of women in LMIC and uncover what specific digitally collection forms can tell us about the day-to-day lives of mothers.

## Methods

Data came from the pilot study, Sensing Technologies for Maternal Depression Treatment in Low Resource Settings (StandStrong) [23]. The development, pilot protocol, and feasibility findings of StandStrong are described in detail elsewhere [24, 25] and is registered with an International Registered Report Identifier (IRRID): DERR1-10.2196/14734. Below, we describe the elements specific to this analysis.

### Setting

This study was conducted in Chitwan, Nepal a diverse southern region of Nepal that borders India. The region is predominantly agrarian, but also contains Nepal’s fourth largest city. Chitwan was selected due to longstanding relationships with the local government and health system. The average age of marriage is 17.9 and the average age for first childbirth is 20.4. The legal age of marriage is 18, however, 52% of women are already married by this time (Ministry of Health—MOH/Nepal & ICF, 2017).

### Study population and sampling

This study was implemented between November 2018 through April 2019. Young mothers were recruited at health posts during monthly infant vaccination days. Women were included if they were 15–25 years old and had an infant between 1–12 months old. All mothers were administered depression screening using the Nepali validated PHQ-9 [26]. The Nepali PHQ-9 has a cut-off of 9 (sensitivity of 94% and specificity of 69%), with a positive predictive value of 0.33, and negative predictive value of 0.99. For non-depressed, a cut-off of less of 7 has a sensitivity of 100%, specificity of 55%, PPV of 0.26, and NPV of 1.00. This means that an adult presenting to primary care centers with a score of below 7 has an extremely low probability of having depression (i.e., low probability of being a “false negative”). In this study, we excluded mothers with PHQ-9 scores of 7 and 8 from either group because of greater risk false positives. Following written informed consent, family consent was obtained in a visit to her home. To maximize confidentiality, the participant and her family were trained in data storage on the phone so they could review and delete any information if they chose. The app also had a “privacy timer” allowing mothers to pause data collection for as long as they wanted. This and other integrated privacy practices are described in more detail in the team’s publication outlining the feasibility and acceptability of the passive sensing suite. [24] A description of the recruitment procedures and participation are also provided in detail in Maharjan et al 2021 [24]. In brief, we screened 782 mothers, of whom 320 were eligible for inclusion based on age criteria of the mothers and infants. We screened the eligible mothers for depression. Approximately 92% of the eligible mothers scored below the depression cut-off of 7 (n = 294) and 8% scored above the cut-off of 9 (n = 26). The mixed group scoring 7 or 8 were excluded from the current study because of greater risk of false positives. We created a roster of all eligible women, and, due to limited device availability, were able to collect sensing data on six women at a time. Therefore, we serially recruited non-depressed mothers until we reached our target sample. Two-thirds of the non-depressed mothers and their families that we serially reached out to provided consent and were recruited. We stopped recruitment once we reached our final sample goal of 27 non-depressed mothers. All mothers subsequently screened, who scored below the depression cut-off, were not included in the study. The larger umbrella study recruited both depressed and non-depressed mothers, consenting 27 non-depressed mothers and their families for both passive sensing data collection and qualitative interviews. For this analysis, only mothers that screened negative for depression were included (n = 22) in order to establish an understanding of the daily lives of psychologically healthy young mothers to use as a baseline for future comparison with depressed mothers. We took an iterative approach to qualitative data collection, assessing code and meaning saturation following each interview as outlined by Hennink et al 2017 [27]. By the 22^nd^ qualitative interview, we obtained rich information, saturating our categories of interest, and were confident the information received from the additional 5 non-depressed women are well-represented in the current interviews.

Ethical approval was received from the Nepal Health Research Council (#327/2018) and George Washington University Institutional Review Board (#051845). Written informed consent was garnered from participants over 18 years. For participants under 18 years, written assent and parental written informed consent from participants under 18 years was obtained. Verbal informed consent from adult members of their household was also obtained. All participants agreed to have their data included in relevant publications and presentations of results, including direct quotations.

### Data collection

The procedures and results described here refer to Component 2 of the original study protocol (Poudyal et al., 2019), including only non-depressed mothers. Study enrollment lasted for 14 days. For passive data collection, mothers were given a low-cost android smartphone and a Bluetooth beacon that attached to her infant’s clothes. A study team member briefed each mother and her family on the technical use of the phone and beacon and made regular phone calls and household visits to ensure the technology was working, answer questions, and conduct qualitative interviews. On average, mothers were visited by a study team member five times throughout the two-week data collection period.

#### Passive data collection

The devices used in this study (Samsung J2 android smartphone and Bluetooth RadBeacon dot) were selected following extensive ethnographic inquiry regarding acceptability and feasibility in the study site [18]. Specific technical details of the technology package and its operation are reported elsewhere [28]. Briefly, the Bluetooth RadBeacon dot was developed by Radius Networks and attached to a belt around the infant’s torso. Mothers were instructed to carry the smartphone with them throughout the day and given a cell phone pouch in case no pockets were available. This analysis leveraged three types of data obtained from the devices: beacon proximity, physical activity, and geographic location. A custom-built Electronic Behavior Monitoring app (EBM version 2.0) was developed to capture this data passively every 15 min between 4 am and 9 pm. Table 1 gives a detailed overview of the passive sensing domains and data collection methods.

**Table 1:** Passive data collection methods.

Domain	Description	Devices/Tools	Passive sensing data
Physical activity	Time spent inactive, standing, walking, on vehicles, etc.	Samsung J2 Android smartphone	Accelerometer data from smartphone with mother
Geographic movement	Range and location of daily movement in community	Samsung J2 Android smartphone	GPS data from smartphone with mother
Mother-child interaction	Daily consistency of mother-child routine	Samsung J2 Android smartphone	Mother-child proximity measured between smartphone with mother and passive Bluetooth beacon attached to child
		Bluetooth beacon (RadBeacon dot)	

#### Qualitative data

Three types of qualitative data were collected: in-depth interviews, daily diaries, and field notes written by the research assistants following participant encounters. Two in-depth interviews were conducted with mothers. The first was on the second or third day following the acquisition of technology. This interview elicited details about her experiences related to motherhood, child rearing, her daily activities, her relationships, and psychosocial wellbeing. This interview lasted approximately 40 minutes. The second interview was conducted on the last day of data collection (Day 14) and elicited her and her family’s experiences with the technology package and data collection process. The interview guides can be found in the supplementary material. On the penultimate day of data collection, mothers completed a structured daily diary recounting her activities on the previous day. This included her main activities every hour between 5am and 9pm, where she was located, where her child was located, and who else was with her. It also elicited information on her waking time, nap times, bedtime, and breastfeeding. Finally, field notes were written systematically following every participant encounter and documented the mother’s environment and information that may not be captured by the transcript. This included histories provided by the mother in conversations before and after the interviews, interruptions, emotions, and what happened during silent periods. It also included information gleaned during phone call check ins. An advantage of the intensive and frequent data collection procedures was that study team members established close rapport with both the mother and her family due to multiple encounters. This increased participant comfort, additional opportunities for her to share her experiences, and allowed for detailed field notes. We saturated our eligible sample (e.g., all women were interviewed) and transcript review triangulated with field notes indicated no new themes were emerging at the end of data collection. The Consolidate Criteria for Reporting Qualitative Studies (COREQ) checklist provides more details on the qualitative data collection procedures and is included in Supplementary material.

### Data analysis

#### Passive sensing data analysis

Sensor data aggregated by the EBM v2.0 app was downloaded from the smartphone, exported in a csv file, and pre-processed and cleaned on import into a SQL database. These data were then loaded into a JupyterLab Notebook. The proximity data collected from mother’s phone and child’s proximity beacon were collapsed for hour intervals such that, any 15-minute scan that detected a beacon within an hour was coded as ‘with the child’ and if all four readings did not detect a beacon, the reading was recorded as ‘away’. The result of the query was either present or not present for each hour interval between 4 AM and 9 PM. Similarly, the activity data were processed in the same way so that any ‘movement’ (vehicle/bicycle/on-foot) captured by the phone was categorized as moving. Using scikit-mobility, GPS data were loaded into a trajectory DataFrame. Data were pre-processed to remove noisy points (if the speed required to move from point to point was greater than 500km/h), and compress lat/lon pairs with 100m of each other while retaining the trajectory structure of the data. Several mobility measures were then applied to these data including max distance from home (great-circle distance between two points), and radius of gyration (characteristic movement range each day) [29]. GPS heatmaps were created using the heatmap function in scikit-mobility to plot visited points on a Folium map and visualized using OpenStreetMap^®^ [30]. Graphic representation of the dichotomized proximity and activity data by hour (aggregated across all 14 days of data collection) were produced for each woman. Women that had fewer than 100 data points for each passive sensor across 14 days of data collection were not included in a given domain because the significant amount of missing data made comparison with observed qualitative trends impractical.

#### Qualitative data analysis

All in-depth interviews were conducted by bilingual (Nepali and English) female research assistants, audio recorded, and transcribed verbatim. One translator then translated Nepali transcripts into English, ensuring culturally meaningful terms were preserved in their original language. These terms were given an English term derived from a standardized Nepali mental health glossary specific to emotional and psychological terms (https://bit.ly/2IQgY2X). All fieldnotes were combined with the transcript text and uploaded into Nvivo 12.0. Daily diaries were also uploaded into Nvivo and tagged with the appropriate participant so data could easily be triangulated with transcripts and field notes. The mother’s demographic data (age, parity, and household members) were uploaded into Nvivo as attributes. Analysis took a systematic thematic approach [31]. First, a codebook was developed combining both deductive and inductive themes expected to explain passive sensing data (transportation, isolation, leaving home, childcare support, household activities, etc.). Open coding was used to develop inductive themes from the data (e.g., cultural practices) that also appeared to explain passive data patterns. Once the initial coding scheme was finalized, a subset of transcripts were coded by three members of the study team to determine inter-rater reliability. Disagreements were discussed, codebook adjusted, and another batch of transcripts were coded until a kappa of 0.7 or higher was achieved. All transcripts were coded and thick descriptions were developed to summarize the depth, breadth, and nuance of each domain as they related to each piece of passive data (activity, proximity, and geospatial movement) [32]. Daily diaries were triangulated with the passive data patterns and incorporated into thick descriptions. We describe the results below, first highlighting the dimensions of women’s lives that may explain passive data patterns and then providing participant illustrations of how passive data profiles might illuminate important aspects of young mothers’ psychosocial experiences.

## Results

The sub-sample for this analysis consisted of 22 young mothers ranging from 16 to 25 years of age with infants averaging 5.7 months old. For 81% of women, this was their first child. The majority (59%) were from indigenous ethnic groups (Janajati/others). Most of the women were Hindu (77%). Nearly 91% of the sample had a secondary grade education or less. The majority of women were full time mothers, assuming the majority of childcare and household work, and about 23% had formal employment outside the home. Table 2 contains details of this demographic information. For activity, five women (P22, P43, P49, and P52) were excluded for having less than 100 readings. For proximity, three women were excluded for having less than 100 readings (P43, P49 and P52). For GPS, eight women were excluded for having less than 100 hours of GPS data (P6, P22, P24, P26, P28, P49, P52, and P53). Daily diaries were not collected for two participants (P1 and P20). For passive sensing data, 48.1% of activity data were collected (3,304 readings for the total sample); 43.9% of proximity data (3,087 readings); and 36.7% of GPS data (2,527 readings) were available for analysis. Across participants, there is variation in engagement primarily due to phone battery charge challenges with access to electricity, data usage exceeding prepaid limits, and the burden of carrying the mobile phones at all times. Detailed findings regarding use, acceptability, feasibility, and limitations of the passive data strategy among this sample of young mothers are reported separately [24]. Overall, and vis-à-vis other pragmatic passive sensing studies, we obtained relatively strong data capture, particularly for the low-income context [33, 34].

**Table 2:** Demographic characteristics of study sample.

Total participants (n = 22)	N (%) or mean (range)
**Mother age**	**19.6 (16–25)**
16–18	6 (27.3)
19–21	11 (50.0)
22–25	5 (22.7)
**Who helps with caregiving during the day?**	
No-one	4 (18.2)
Other family members	18 (81.8)
**Nuclear household**	
Yes	5 (22.7)
Caste*	**N (%)**
Brahmin/Chhetri	5 (22.7)
Janajati/Other	13 (59.1)
Dalit	4 (18.2)
**Religion**	**N (%)**
Hindu	17 (77.3)
Buddhist	3 (13.6)
Christian	2 (9.1)
**Education**	**N (%)**
Primary	3 (13.6)
Secondary	17 (77.3)
Higher	2 (9.1)
**Child gender**	**N (%)**
Female	11 (50.00)
**Child age**	**5.77 (2–12)**
1 to 7 months	17 (77.3)
8 to 12 months	5 (22.7)
**Number of children**	**1.18 (1–2)**
First child	18 (81.8)
**Participant Occupation**	**N (%)**
Full time mother	17 (77.3)
Agriculture	3 (13.6)
Business	1 (4.6)
Day wage laborer	1 (4.6)

**We use caste here to denote the Hindu-constructed class system. We have simplified the categories which distinguishes Brahmin/Chhetri (historically religious leaders and warriors), and Dalit (historically manual laborers), and ethnicities that are not classified in the Hindu caste system (e.g., Janajati/other). Some castes and ethnic groups (such as Dalit and Janajati) may face disproportionate inequity due to systemic racism and historic segregation.

### Infant proximity

**Fig 1:**
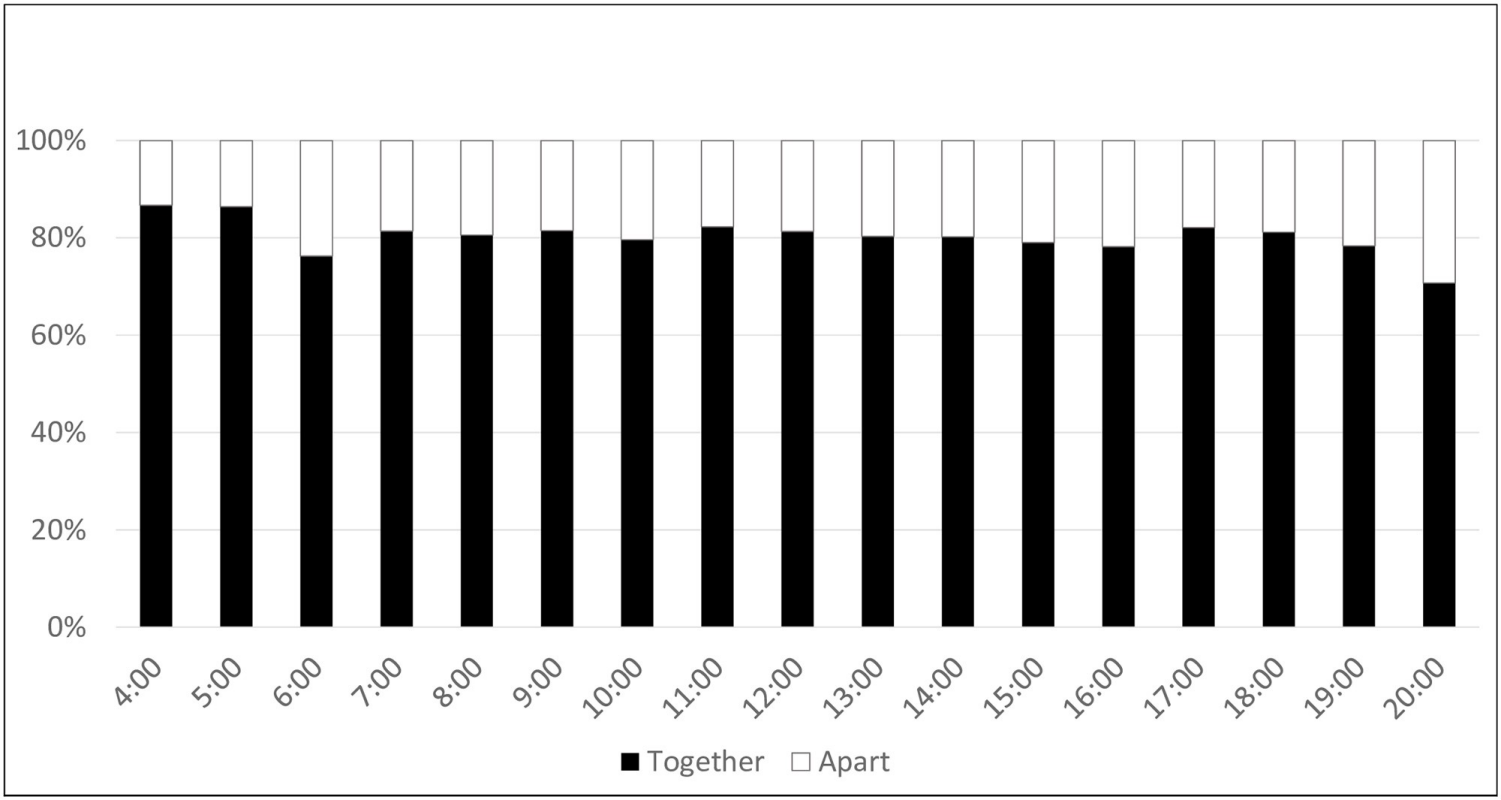
Aggregated proximity by hour averaged across 14 days for all women: Time apart or together (n = 21). Together: Mother’s cell phone detects Bluetooth beacon attached to the infant.

Fig 1 illustrates the average daily proximity pattern across our sample. Mothers were near their infants, on average, 81% of the day (4am to 8pm). Nine mothers were detected to be with their infant over 90% of the period, while two were with their infants for less than 60% of the period. The average daily pattern indicated that mothers were most likely to be apart from their infant early in the morning (around 6am) and late in the evening. Women broadly fit within three categories of child proximity patterns, those together nearly all day, those apart more than average, and those episodically apart at the extremes of the day. We describe these patterns, triangulated with women’s descriptions of their everyday lives to provide various explanations of these findings.

#### Average proximity patterns of mothers

Every mother described her role of caretaking and being physically with the infant as her primary responsibility, one that fundamentally brought happiness and joy. Nearly all the women described the largest change they experienced after becoming a mother was having to put their baby before their own needs and desires. Women reported that they must remain with their infants ‘all day’ to provide important care. Often, this proximity was cited as a proxy indication for ‘good mothering’. Mothers described two main drivers bringing them close to their child (1) instrumental care, including breastfeeding, bathing, giving an oil massage, etc.; and (2) emotional care (playing, consoling, teaching, etc.). Mothers placed an emphasis on maintaining her infant’s hygiene (n = 16) and, as such, bathing her child, changing diapers, and cleaning her infant’s clothes took up substantial portions of her day. Instrumental cleaning activities might have taken the mother farther from her infant (water taps or water containers were kept outside the home), and women did this while their child was sleeping (which may indicate why levels vary slightly throughout the day. Similarly, women that were exclusively breastfeeding, especially those with children under 7 months, indicated that they were doing so several times throughout the day and was described as a reason why mothers had to be close to their infants throughout the day, preventing them from going ‘anywhere she wants.’ The need to be physically close to her infant was one of the most commonly noted transformations following new motherhood—some women indicated this as a lack of freedom, others as a ‘responsibility.’ Indeed, one mother mentioned ending her educational pursuits because she had to breastfeed her baby (P23). Other women (P7, P51) stated that they struggled to get rest during the day because they had to look after their children without support. These instrumental tasks could be overwhelming given the amount of effort and time (changing clothes after every pass of urine).

Emotional care was the second most reported theme that brought mothers close to her child and occurred throughout the day. This usually took the form of playing with the baby, talking with the baby, carrying the baby, and soothing. Mothers placed an emphasis on demonstrating love and affection for their child in addition to providing them with instrumental care. In several cases, mothers announced that engaging in emotional care activities with their baby made them happy. Additionally, in more than one instance, mothers reported that they played with their babies as a way of taking care of themselves. Mothers may have enjoyed providing emotional care to their child because it provided them with an opportunity to get to know their baby more, unlike when they provide instrumental care, which focused on fulfilling the baby’s physiological needs.

**Fig 2:**
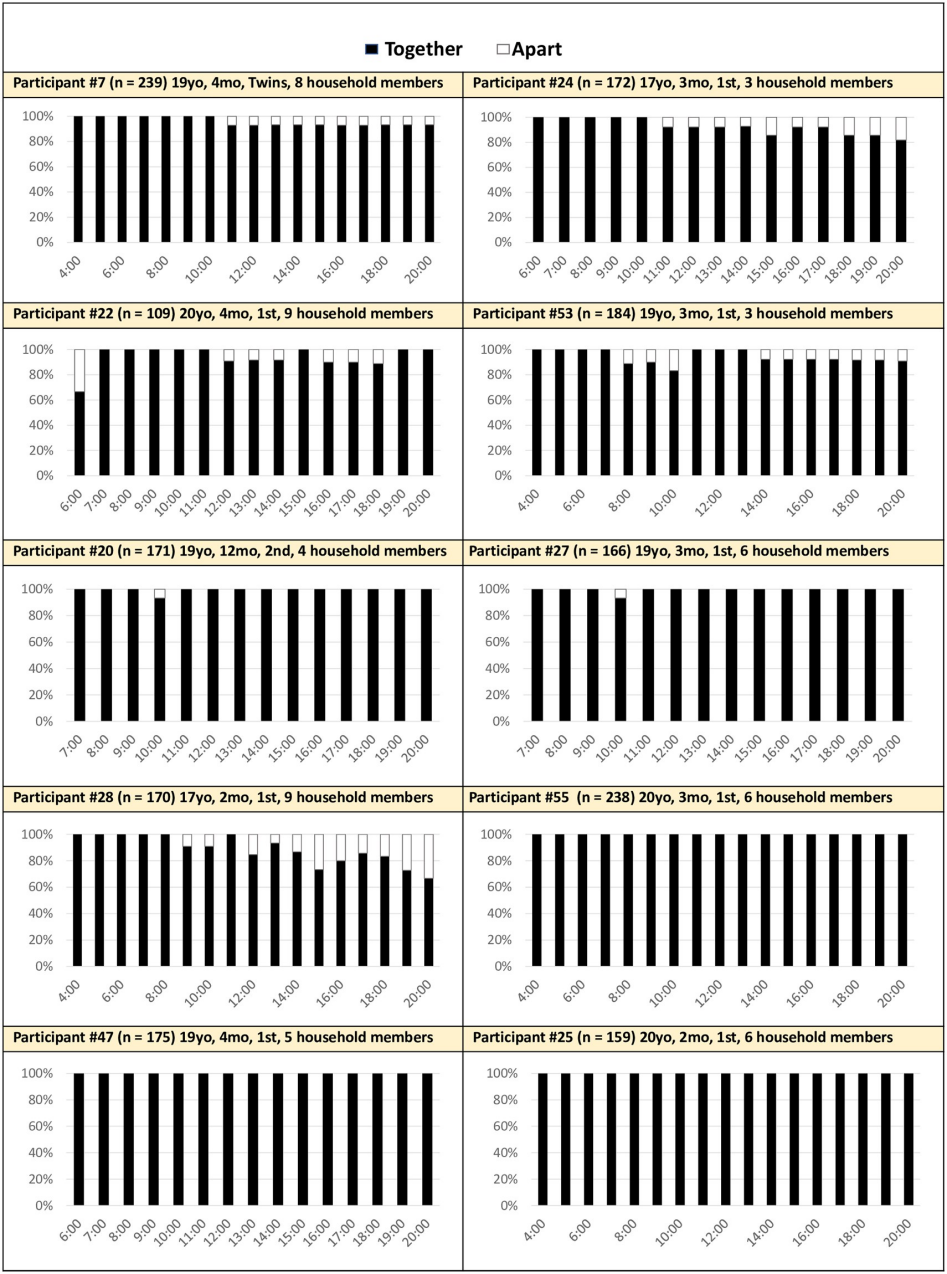
Proximity by hour averaged across 14 days: Mother-infant together all day (n = 10). Together: Mother’s cell phone detects Bluetooth beacon attached to the infant.

*Together all day*. Ten women (48%) were together with their infant nearly the entire day, with only episodic and limited instances apart from their child (Fig 2).

Nearly all the mothers in this group were first time mothers with younger children (2–4 months). Younger children required more frequent and intense instrumental care. Mothers noted several emotions connected to her physical proximity with her infant, including both positive attachment, fear, and stress. Participant 47 smiled as she described the time she spends with her daughter. She said, “*She sleeps well and I give oil massage and sekne [physical warmth]…babies don’t cry if we perform these types of activities and play*. *Playing and laughing is good for their health*.” She went on to describe that her family helped. For example, her husband held the baby while they ate dinner together. *“In this way*, *he cares for me*.” On the other hand, the demands and uncertainties of new motherhood brought negative feelings. For example, participant 28 described that she has to let her baby cry alongside her so she can do her work, “*After I finish all my household work*, *I stay in my room with my baby*. *I have to look after her*. *I don’t have time to think about me*, *she doesn’t let me wash my own clothes—at that time*, *I feel difficult*.” Other women echoed the stress of completing their household chores with their child alongside them, wherein managing the demanding emotional care on top of cooking and cleaning the home was overwhelming.

These women also described limited household help. For some, this was because they lived in a nuclear home, such as participant 20 who was 15 when she had her first child and lived in a nuclear household, where her husband workd all day outside the home. For others, they may have lived in a multigenerational household, but did not easily receive help. Without childcare assistance, these women needed to be with their child throughout the entire day, bringing them with them as they did their other daily tasks. Participant 27 described her day, “*I just stay with him and hold him…*.*I feel shy and scared [to ask my mother-in law for help]…I am scared [my mother-in-law] might scold me (gali garnee)*.” Women’s relationship with household members, particularly in-laws, seemed to contribute to her spending time physically distant from her baby. Notably, when women were apart from their child in this group, it was to do another household chore leaving little time for ‘themselves’.

**Fig 3:**
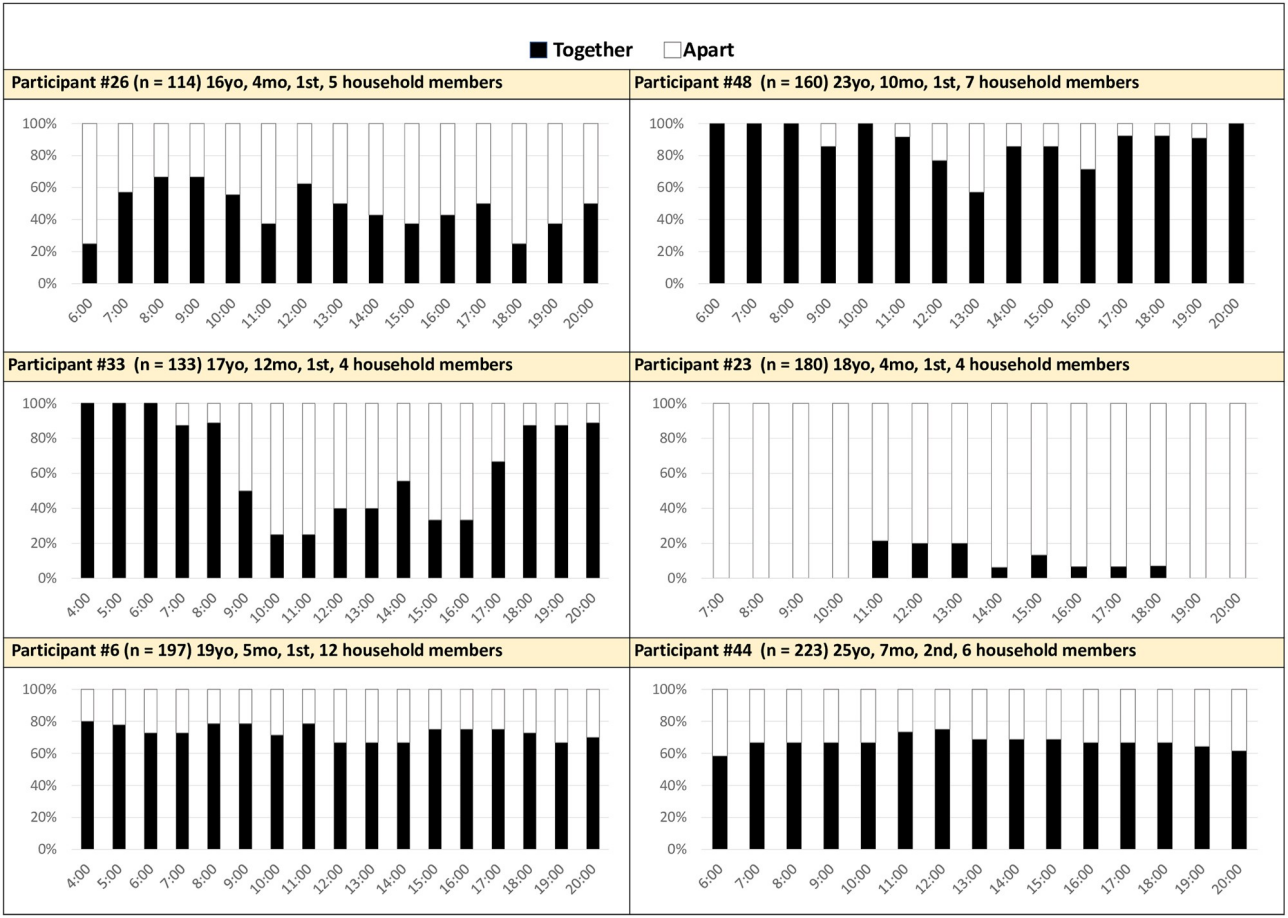
Proximity by hour averaged across 14 days: Mother-infant apart more than average all day (n = 6). Together: Mother’s cell phone detects Bluetooth beacon attached to the infant.

*Apart more than average all day*. Six women (29%) were apart from their children much more than average throughout the day. Some women were continuously apart, and some were apart during certain periods of the day. Proximity graphs of these women can be found in Fig 3.

Three women in this group were in the formal workforce and they did not bring their children with them (P33, P44, P48). This mapped onto their beacon data. For example, P33’s beacon was not detected often between nine am and five pm, her typical workday. She is a daily waged labor worker and left her 12-month daughter with her mother and brother during the day. Her husband worked abroad, and, because of her love marriage, she remained in her maternal home (due to resulting tension with her husband’s family) and was happy to have the support of her own family. For mothers that must be away from their children for work, finding familial support was essential. Some mothers were unable to pull on their household members for help. For example, Participant 6 lived with 11 other family members in her husband’s home but was uncomfortable asking for help and needed to leave her baby to do required tasks. She explained her overwhelming responsibilities, “Still she is small, at this time I have to look after her when she starts crying. When I am free, I can look after her but when I remain busy it would be better if someone else could. I can’t force other people to look after her. If other people didn’t give attention towards my baby at that time, I myself have to manage all these things…They [in-laws] don’t even talk to me…”. Formal work and household support were integrally related to a mother’s proximity to her child throughout the day. Two women in this group qualitatively reported usually being together with their infants (P23 and P26), indicating disagreement with the beacon data.

#### Apart more than average in morning and evening times

**Fig 4:**
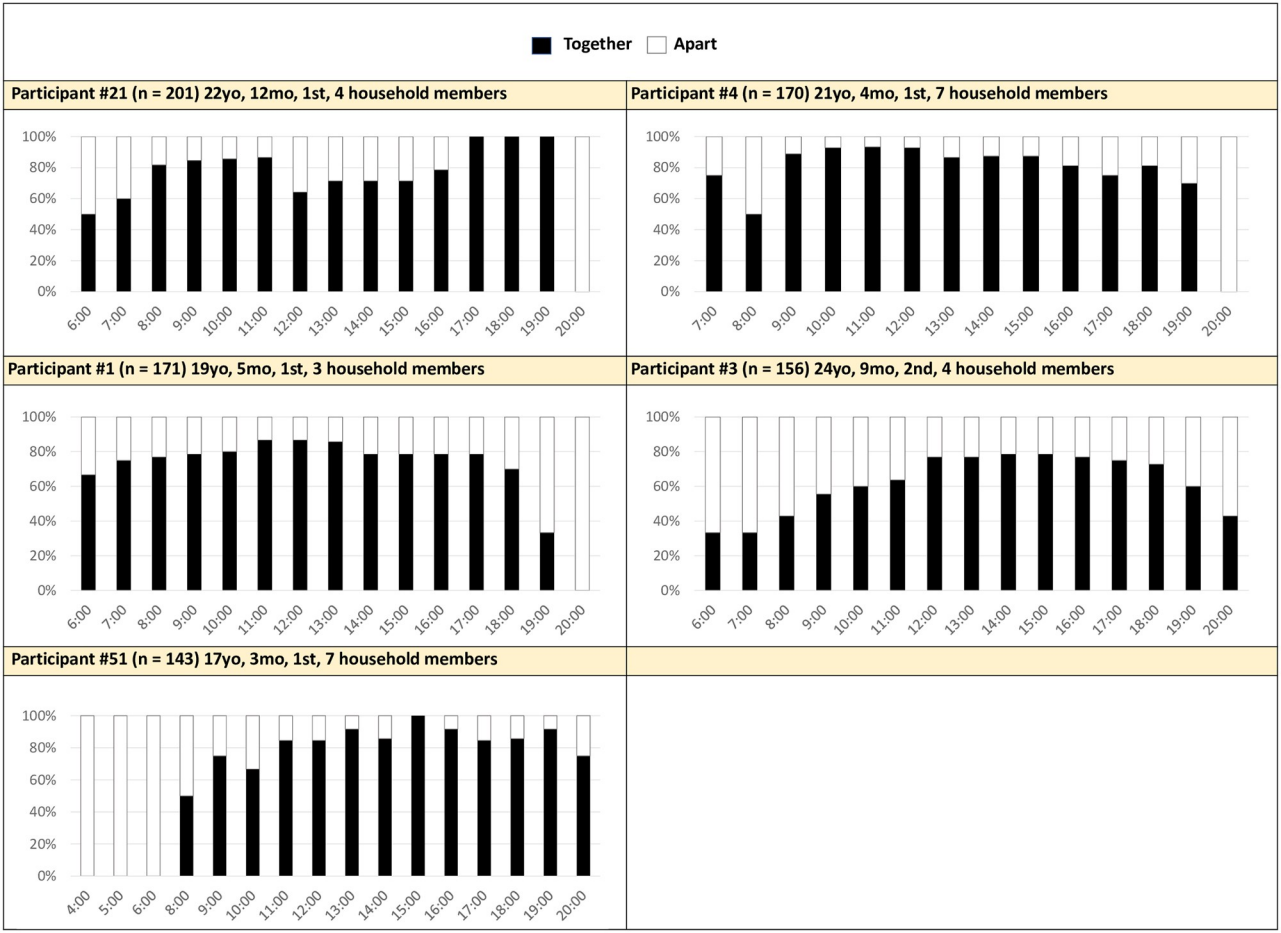
Proximity by hour averaged across 14 days: Mother-infant apart more than average in morning and evening times (n = 5). Together: Mother’s cell phone detects Bluetooth beacon attached to the infant.

Five women (24%) were apart from their children more than average in the mornings and/or evenings. Most of these women were usually with their child in the middle of the day, though P21 was also apart from her child often for a period of the afternoon. The graphs of P1, P3, P4, P21, and P51 are included in Fig 4.

Women in this group often did chores first thing in the morning, while their child was sleeping. This may explain why women were often not with their child in the early morning. Cooking and cleaning typically happened outside the home in the open air. In her daily diary, P51 described her morning routine beginning at 6am. She swept the yard around the house and prepared the morning meal for her household. Her three-month-old daughter stayed with her husband in the bedroom until 9am. Similarly, P3 reported that her responsibilities had increased dramatically since the birth of her second child. She had to cook a separate meal for her daughter and wash all their clothes. She did this outside while her daughter remained in the bedroom with her husband. In her daily diary, she noted she was busy doing chores every hour until the early afternoon, then started cooking and cleaning again towards the evening. She exclaimed, “A mother has to face many difficulties. And we must face *all* these difficulties.” These women were typically able to leave their children with other family members so they could perform their household responsibilities, on top of caring for their children.

### Hourly activity

**Fig 5:**
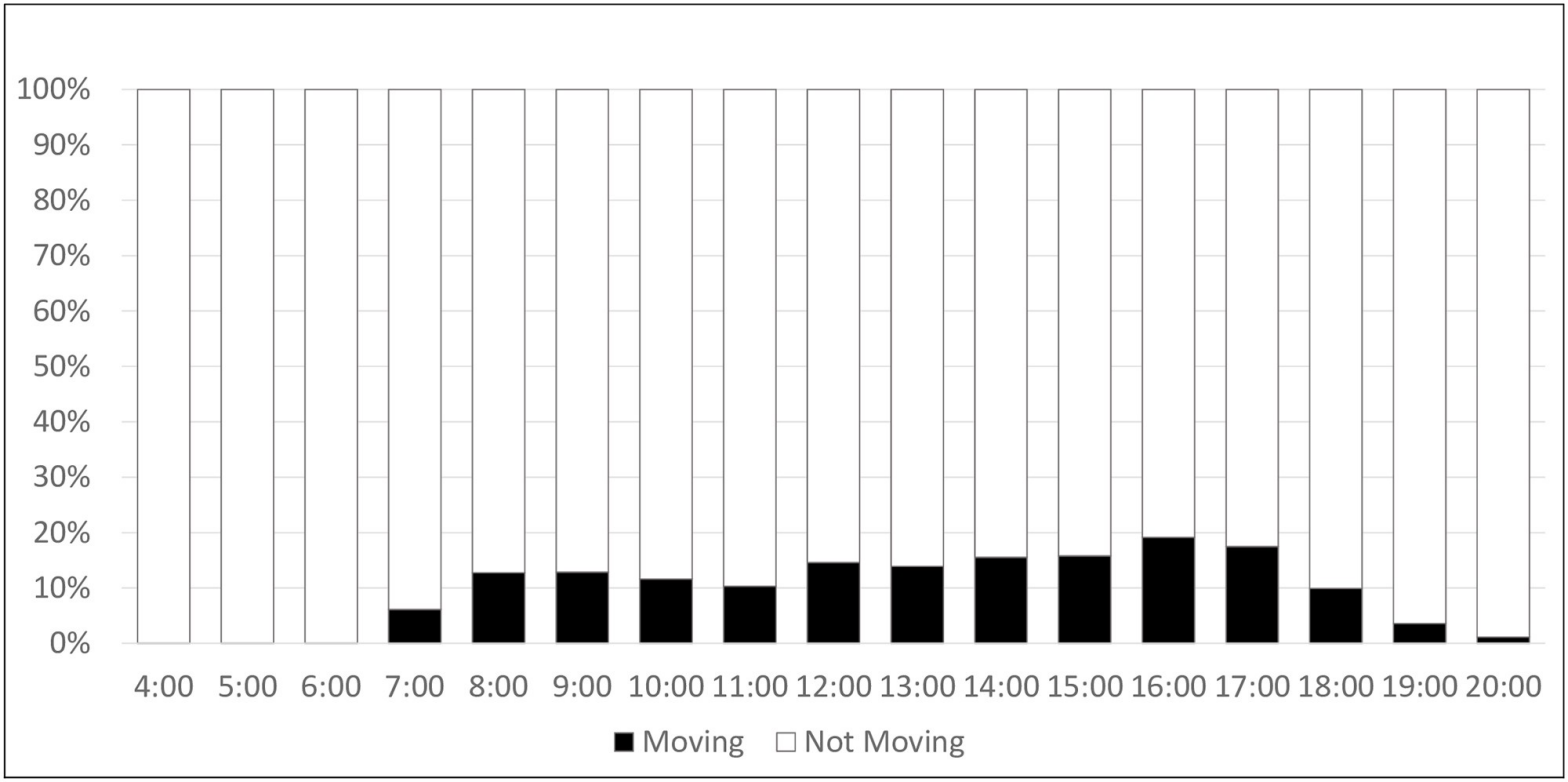
Aggregated hourly activity averaged across 14 days for all women (n = 16). Activity: Movement detected by the accelerometer within the mother’s cell phone (e.g., walking, cycling, riding a vehicle). No movement was identified if the phone detected sitting or still positions.

Fig 5 illustrates the average hourly activity of mothers throughout the day, as measured by phone accelerometer data. Generally, mothers were active around 10–20 percent of the time in any given hour. Average detected hourly activity spiked from 5 percent during the 7 a.m. hour to nearly 15 percent during the 8 a.m. hour when most mothers were up and completing their daily tasks. Detected hourly activity remained at this level during the 9 a.m. hour and then slowly decreased from 10 a.m. to 1 p.m. when mothers were usually sitting down for a meal or taking a midday rest. Average detected hourly activity rose back up to nearly 20 percent from 2–4 p.m. when mothers usually resumed their daily tasks and began to drop off once again from 5 p.m. and onward. Mothers were grouped into three clusters of hourly activity level. Mothers who were “very active” experienced peaks in movement that considerably exceeded 20 percent (n = 4). Mothers who were considered “moderately active” were active around 20 percent of the time during peak activity hours (n = 5). Mothers considered to be “largely inactive” had very low or no activity peaks throughout the day (n = 7). During hours when they were active, peak movement was only around 10 percent.

#### Very active

**Fig 6:**
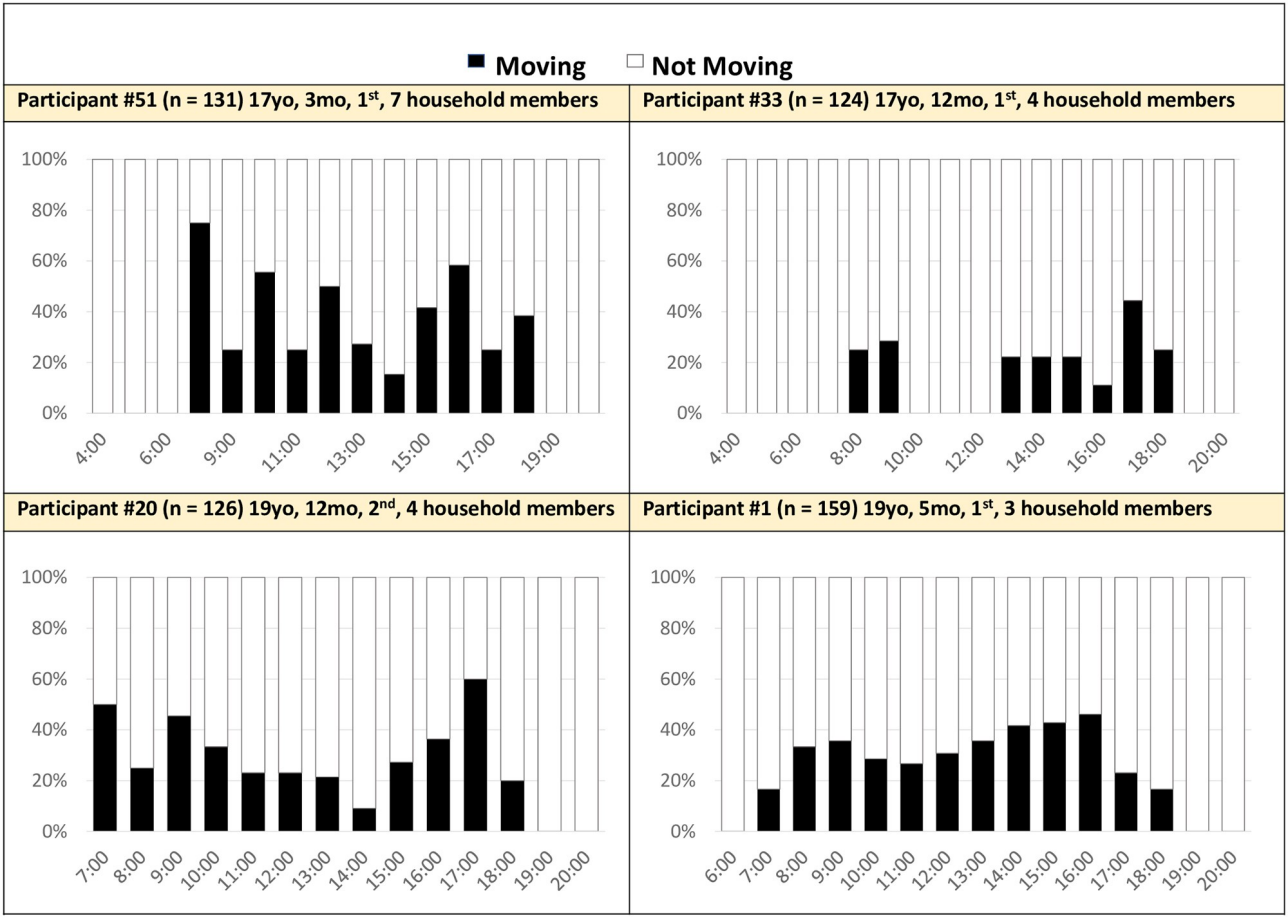
Hourly activity averaged across 14 days: Very active mothers (n = 4). Activity: Movement detected by the accelerometer within the mother’s cell phone (e.g., walking, cycling, riding a vehicle). No movement was identified if the phone detected sitting or still positions.

Four women (25%) had much higher hourly activity detection than average throughout the day, with multiple peaks that significantly exceeded 20% (Fig 6). Some were continuously active, while others were active episodically.

Housework largely drove their increased detected hourly activity. All four highly active mothers shared they did extensive housework in the morning (e.g., sweeping), explaining the heightened activity witnessed around 7–8 a.m. Mothers also shared that they spent the day playing and carrying their baby, highlighting that movement and actively holding them calmed them down. Common household chores were labor intensive, including cleaning their home, sweeping in and around the house, washing dishes and clothes/diapers. Washing clothes was especially labor intensive for women in rural Nepal given the lack of running water and the amount of dirty clothes infants produce. Mothers assumed sole responsibility for all childcare activities, prompting expressions of frustration with the growing load of work. Participant 51 generalized her difficulties with other mothers in her community, “We have to check whether they pass urine or not, change their clothes at night, we have to regularly breastfeed, we have to help them sleep at night. These types of difficulties mothers have to face.” Despite living in a large household, she did not have substantial childcare or household support. Indeed, mothers stated they could not accept childcare support. For example, Participant 1 shared she did not let anyone care for her baby other than her, saying, “Because I can’t trust other people easily. I think they might drop my baby while walking or doing some work. I think other people don’t provide care like I can. So, I take this baby all the time with me. I don’t leave her with other people.” Alternatively, mothers who worked paid jobs usually had childcare support while they were working, but still may have had high hourly activity levels. Participant 33, for example, did labor work during the day such as carrying bricks, cement, and sand, requiring substantial physical activity and movement. Given these mothers’ experiences, a combination of childcare/play, housework, and labor work in the context of limited childcare support increased detected hourly activity levels.

**Fig 7:**
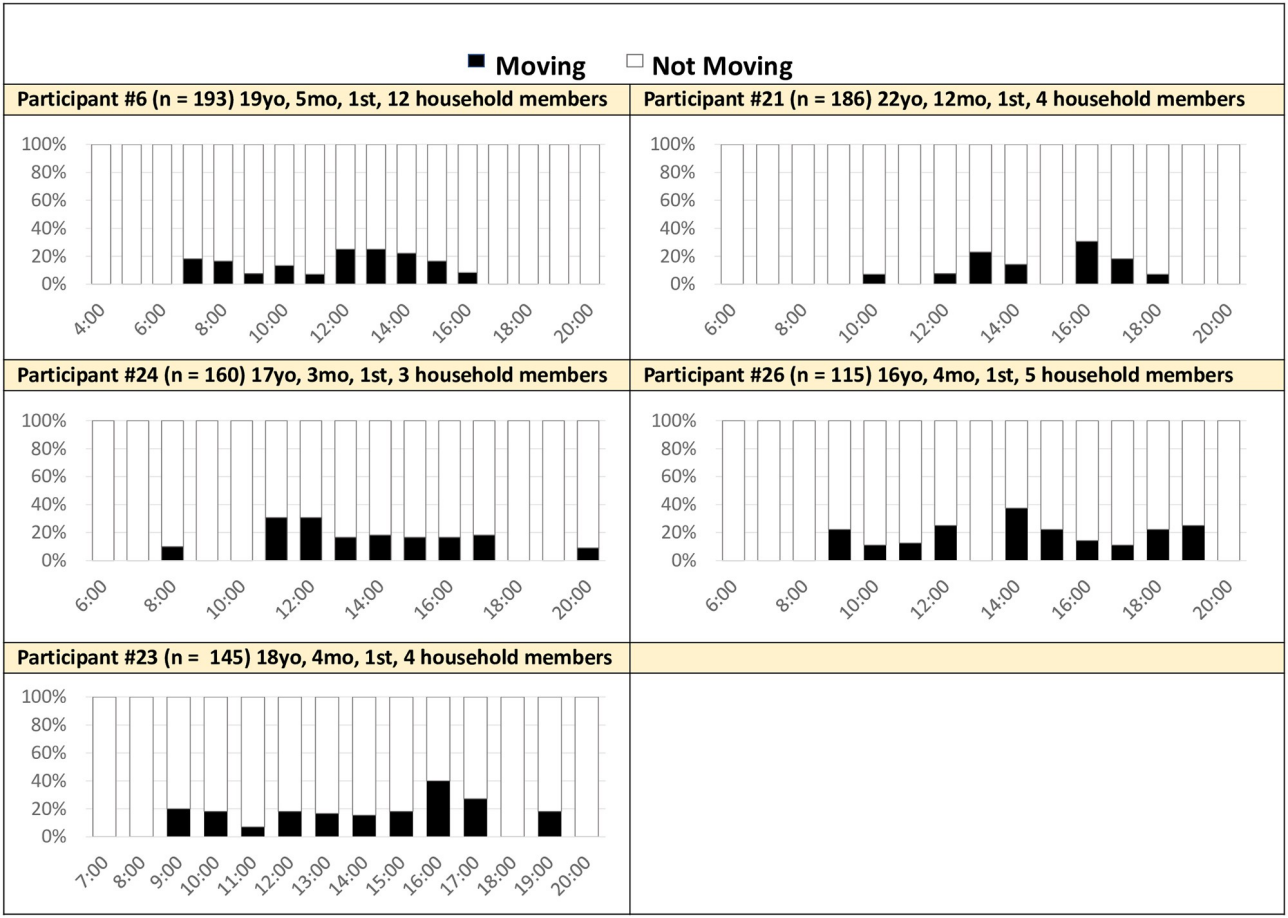
Hourly activity averaged across 14 days: Moderately active mothers (n = 5). Activity: Movement detected by the accelerometer within the mother’s cell phone (e.g., walking, cycling, riding a vehicle). No movement was identified if the phone detected sitting or still positions.

*Moderate hourly activity*. Five mothers (31%) were considered “moderately active”. They were usually active in the middle part of the day and during peak activity, they were active around 20% of the time (Fig 7).

Similar to “very active” mothers, mothers with moderate hourly activity levels still had a considerable amount of household work to complete throughout the day. However, many of them reported having some support throughout the day, absolving them of some of the more movement driven household activities. Participant 23 received help from her husband and mother-in-law and shared that she found it difficult to complete her housework and watch her baby when she did not have that support. For example, she felt difficult one day when her husband and mother-in-law were not home because, “On that day, I have to do household work also. So, I feel it is difficult to look after my baby and do all the household work.” Mothers shared that specific responsibilities might be shifted to other household members, but still reported other, perhaps less ‘active’, responsibilities around the clock.

Another possible reason why these mothers had less detected hourly activity than the four mothers above is because they spent more time breastfeeding. These women described breastfeeding as sedentary, sitting and holding their babies. While all the “very active” mothers mentioned breastfeeding in their interviews, some mothers in the “moderate hourly activity” category spoke about having to breastfeed multiple times a day. For example, Participant 23 shared she had to stop attending college because she needed to breastfeed her baby so often, saying, “Because I have to breastfeed [many times], he used to cry if I leave him for long time.” Participant 26 also shared she breastfed as a way to pacify her child. All of these mothers had very young babies (under 4 months old) that likely needed to breastfeed often for longer periods of time compared to older babies.

**Fig 8:**
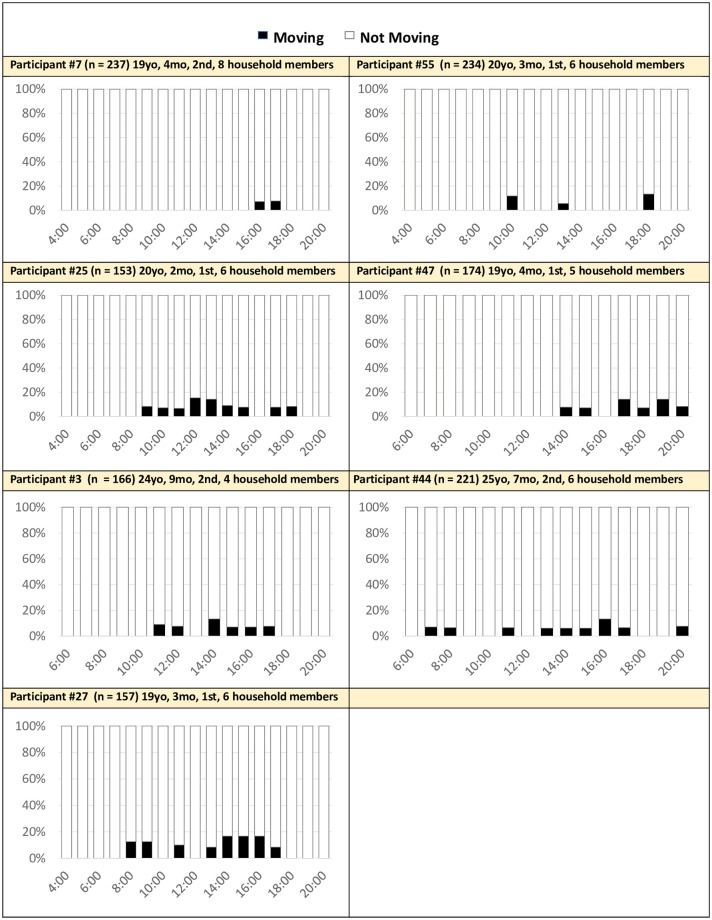
Hourly activity averaged across 14 days: Largely inactive mothers (n = 7). Activity: Movement detected by the accelerometer within the mother’s cell phone (e.g., walking, cycling, riding a vehicle). No movement was identified if the phone detected sitting or still positions.

*Largely inactive*. Seven mothers (44%) were considered “largely inactive” (Fig 8).

Of these seven mothers, almost all of them came from larger, non-nuclear households meaning they were more likely to share the housework with other family members. Two of these mothers (P44, P55) expressed that they experienced a decrease in household chores after they became mothers. For example, Participant 55 said, “*Before giving birth to my baby I used to prepare meal but now I don’t*. *I used to wash my husband’s clothes*, *but now I don’t*. *Now*, *I only wash my baby’s and my clothes*. *I used to clean whole house*, *but now I clean just my room*.” These mothers still indicated that they were busy the whole day, with no time for themselves.

Another similarity within this group is nearly all the mothers had young babies under seven 7 months. Mothers explained that younger babies demanded more sedentary caretaking (e.g., breastfeeding, providing warmth, oil massage, etc.). This, in combination with decreases in housework, likely drove the inactivity being observed in the passive data.

### Geospatial movement

We analyzed the GPS data of 14 mothers with over 100 data points. The mothers were sorted into tertiles based on their radius of gyration, defined as the average radius that a person traveled from their center over the course of a day and results are presented in Table 3. The average radius of gyration among our sample was 1675 meters, and the median was 798 meters. The ranges of mothers in the first, second, and third tertiles were 47–618, 654–1455, and 2047–5100 meters, respectively. The mothers commonly left their homes to visit the following places: health facilities, market/shops, and their maternal homes. Formal work, college, temples, new places, and other errands were mentioned less frequently. Most women only reported leaving their child at home for a few minutes to an hour and a half, but Participant 21 shared that she went to the market and was gone for seven hours. Many mothers expressed that they only left the house for essential reasons, though Participant 1 shared that she liked to visit new places for entertainment.

**Table 3:** 

		1st tertile (46.6–618.2m)	2nd tertile (654.4–1454.6m)	3rd tertile (2047.8–5099.5m)
**Radius of gyration: The** average radius that a person travels from their center over the course of a day	**Participant IDs**	**7**	33	43
		3	**23**	**1**
		**51**	20	21
		**27**	**44**	**47**
		**55**		**25**

Participant ID bolding indicates the infant’s age where bolded IDs are less than or equal to seven months and unbolded are older than seven months.

A few women mentioned other factors besides household chores and childcare responsibilities that keep them at home—for example, Participant 7 shared that concerns for her own health kept her at home. To illuminate the daily lives of women in the lowest and highest tertile, we synthesize qualitative data from interviews, field notes, and daily diaries to understand what brought these women out of their homes and what kept them in their homes.

#### Maximum geospatial movement (third tertile)

The mothers in the highest tertile spent large portions of the day around their home doing housework and taking care of their baby. As mentioned above, women were often kept at home unless they had someone to help them complete their household responsibilities. All of these mothers shared that they had family members who they could ask for help, and none expressed difficulty asking for help. Five mothers shared that their mother-in-law or husband helped with the baby or with chores (P43, P1, P21, P25, P47). Women in other tertiles also mentioned receiving help from family members, but few reported significant help readily offered on a routine basis. Having regularly supportive family members may have contributed to the mothers’ ability to leave the house.

All women in this tertile reported leaving the house without their child at least once. Completing essential errands was one of the main reasons that women left their homes. Three of these women reported leaving their baby with family members to go to the market (P1, P21, P47), and Participant 25 shared that she occasionally left her baby to run an errand at the roundabout. Women in the third tertile ran these errands more frequently than women in other tertiles, who usually reported running errands only on rare occasions. Family visitation was another reason that women left their homes. Two mothers in this tertile reported visiting their maternal home (P47, P1). Young mothers often lived with their in-laws or in nuclear households, so visiting their maternal home was noted as an important source of comfort. Some women also visited other family members—Participant 47 often visited the house of her sister-in-law, and Participant 25 visited her grandmother and her sister. Women with large geospatial movement shared that visiting family members brought them out of their homes, indicating this practice was an important source of connection.

Some of these mothers were accustomed to having more social interactions outside the house before giving birth. Participant 47 shared that she used to go to her sister-in-law’s house before having her baby. Participant 21 used to work in the fields before giving birth. Even in this group of mothers who have large radii of gyration, these mothers shared that they did not travel far because they needed to be near their babies (P1, P21, P25, P47). Participant 21 shared that “*doing oil massage*, *washing clothes*, *taking care of my baby*, *breastfeeding*, *I can’t just go anywhere I want*.*”* Regardless of their volume of geospatial movement, women expressed that the need to be close to their child kept them from travelling very far.

*Limited geospatial movement (first tertile)*. The mothers in the lowest tertile (P3, P7, P27, P51, P55) usually remained very close to home, travelling an average of 47 to 618 meters away from home per day. The age of the baby may have contributed to keeping women at home because women must exclusively breastfeed younger babies. The mean child age in this tertile was 4.4 months, compared to 8.8 and 7.0 months in the second and third tertiles, respectively.

With their household responsibilities, mothers were often kept at home unless they had someone who could watch the baby and/or help with chores. Women with limited geospatial movement did not always have supportive relationships with family members, so they may not have had the help from others that would have enabled them to leave the house. All of the mothers in this cohort shared that they had family members or neighbors who they could ask for help, but only women in this tertile (P7 and P27) reported that it was difficult to ask for help. Lacking supportive family members to help with housework or childcare may have kept women home.

Most women with limited geospatial movement ventured outside of their homes infrequently. Besides a few essential trips outside of the home, four mothers explicitly stated that they usually remained at home all day and felt isolated (P3, P7, P27, P55). This isolation was connected to the motherhood transition. Participant 55 shared that she could not leave the house like she did before giving birth:

“*Now*, *I don’t go for walks*. *I always remain in my home*, *but previously during pregnancy I used to walk here and there*. *I didn’t stay in my home*. *I used to go in my neighbor’s house after finishing my housework*. *Sometimes I went to my aunt’s house*, *sometimes to my maternal home and then come back here to prepare breakfast*. *Then*, *after preparing breakfast again I used to go to my maternal uncles’ home*…*Now*, *I have my baby so I don’t go anywhere*.*”*

Motherhood restricted geospatial movement, limiting valuable social engagement. Mothers articulated that visiting her maternal home was significant for her well-being, but they were either “not allowed’ to return home or were not able given competing responsibilities.

### Participant illustrations illuminating passive data profiles

**Fig 9:**
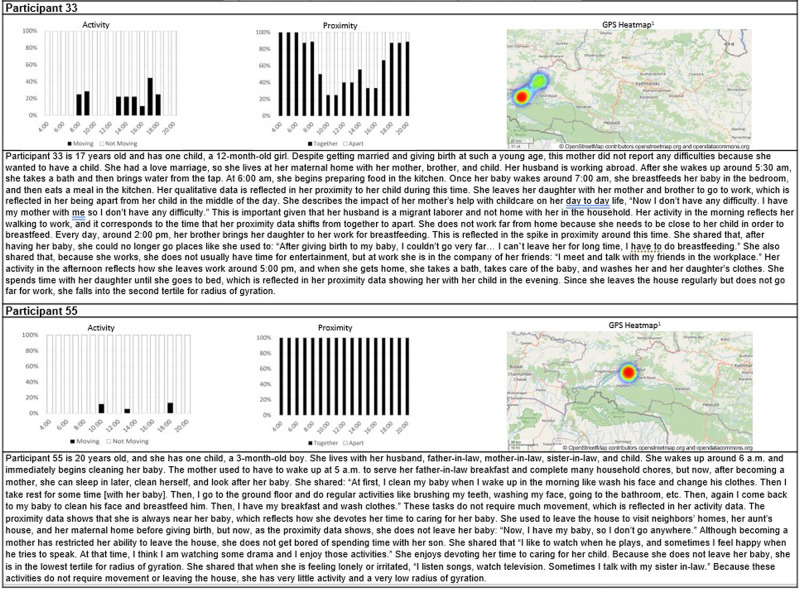
Participant illustrations illuminating passive data profiles. *Red color indicates locations where the index mother was most often captured while green indicates locations where she was less frequently captured. ^1^The image and map data was created OpenStreetMap^®^, an open data platform, licensed under the Open Data Commons Open Database License (ODbL) by the OpenStreetMap Foundation (OSMF). Users are free to copy, distribute, transmit and adapt OpenStreetMap data. More information can be found here: https://www.openstreetmap.org/copyright.

We share participant illustrations of two women to synthesize their passively monitored behaviors with their narrated day-to-day experiences (Fig 9). We combine the activity, proximity, and GPS data of these women to provide a more complete picture of their daily lives and to help understand and interpret the passive data.

## Discussion

### Contribution to mixed methods research

This is the first study to capture various passively collected sensing indicators among young mothers and triangulate their digital phenotypes with qualitative data to gain insights regarding activity and behaviors of typical young Nepali mothers and their infants. Specifically, we highlight that these women have fairly restricted movements, largely influenced by accessible household and childcare support, the age of their infants, and traditional gender and cultural roles. Previous research conducted on maternal movement focuses primarily on demographic, lifestyle, and clinical factors associated with lessened to heightened activity levels [35, 36]. Our research provides novel insights into young maternal activity patterns in the postpartum period and what actions drive various movements (GPS, child proximity, and activity). While there is emerging digital health work to enhance our understanding of human behavior and relationships with digital footprints [16, 37–39], this is the first study to our knowledge that leverages and triangulates multiple methods. Our approach builds on digital integration into maternal health [14, 40] by capturing and increasing our understanding of the lived experiences of young vulnerable mothers.

We found most mothers enrolled in this study upheld traditional gender roles and were responsible for most of the instrumental and emotional child rearing tasks as well as housework. This was not unexpected, given what we know about the cultural expectations for women in Nepal [8, 41]. We found that mothers’ detected activity levels were influenced by their child’s age and the amount of childcare and household support they receive from family and friends. Mothers who had considerable support tended to be more sedentary. Mothers with higher levels of detected activity were less likely to have familial support throughout the day to help them either care for their baby or complete their housework. As a consequence of this, these mothers are usually moving around and working all day, providing them with little time to lay down, take naps, or engage in entertainment.

This constant work schedule coupled with lack of rest time may impact mothers’ health. Many mothers expressed frustrations with their heavy workload and may experience feelings of stress. Some researchers theorize stress can make one more susceptible to both physical and mental health problems [42]. This finding, coupled with proximity and geospatial findings, shine a light on how passive sensing data can be used as part of an intervention supporting maternal mental health.

Mother-infant proximity studies largely focus on household presence or absence of mothers and do not explore more fine-grained distance measures. Previous health studies have primarily focused on the use of interpersonal proximity measurement to assess infectious disease risk [43]. Moreover, relatively few measure proximity to infants specifically. Ozella [44], Kiti [45], and Campbell [46] used infant proximity as a way to understand their social lives (and human exposures) to inform infectious disease transmission and risk. Ozella and Kiti pilot the use of digital sensors, while Campbell uses self-reported paper diaries. All studies identify that the infant spends the majority of time with their mother and focus on the location and specific number of contacts made. None of the studies explore effects of infant proximity on maternal behaviors or psychosocial well-being. To this end, researching mother-infant interpersonal activity through sensing technologies remains novel. Our findings highlight that objective measures of infant proximity give important insights into a mother’s life, including both the benefits and challenges of being near or far from her baby throughout the day. Young Nepali mothers are near to their infants for the majority of the waking day, fulfilling an important social role, offering a rewarding identity (motherhood), and providing positive emotional attachments.

This research also highlights the enormous workload particularly for mothers with limited household support. These concepts have been identified among low-income women with adverse childhood experiences in western contexts [47–49], but limited work is conducted cross-culturally in LMIC [50, 51]. Future investigations can provide more insights into how the transition to motherhood, particularly for vulnerable younger mothers in low-income contexts, affects their daily activities, social world, and subsequent identity. This information can inform behavioral interventions, specifically those focused on psychological well-being. For example, infant proximity patterns can be used to identify need and opportunities for self-care and support, necessary components for maternal well-being.

The geospatial movement data that was collected allowed us to identify groups of women with different geospatial movement patterns. We found that women with lower radii of gyration often lacked support at home. Our future analyses will compare the passive sensing data profiles of the non-depressed women described in this study with depressed women in the same region. To date most studies have evaluated geospatial movement in high-income countries [14], but there are few studies about postpartum women in LMIC that utilize GPS data beyond the purpose of feasibility studies. However, the relatively small distance that the mothers in this study travel (the average radius of gyration is less than 2 kilometers) is consistent with the findings of a study in India that found that, while GPS data indicated that older men travelled less than younger men, older women actually traveled more than younger women because young women with children are largely confined to the household [52]. This limited ability to travel outside the home may have implications on women’s mental health. Interventions that provide child-care or household assistance may enable women to leave their house more frequently, which may have a positive impact on their well-being.

#### Strengths and limitations

This study is the first to deploy a suite of passive sensing data collection approaches in an young maternal population in LMIC. We used an ethnographic and community-based participatory approach to data capture and interpretation by triangulating passive data with qualitative accounts in order to better understand how young mothers describe and explain their everyday experiences vis-a-vis technology based captures of their activities. Multiple encounters between the research team, female informants, and their families, created important trust and several opportunities for data capture across several weeks. Additional remote check-ins by telephone and in-person visits helped to avoid technological barriers and further develop trust with mothers and their families. However, there are important limitations to consider given the novel approach and emerging technology. We describe the feasibility and technical limitations in detail elsewhere [24], but provide particularly salient limitations for this analysis here. First, the phone-based data capture did not detect all activity as the cell phone may have been put down while conducting various household activities, particularly those involving water or threats to the phone’s safety. Data capture only occurred in 15-minute increments throughout the day, limiting the granularity. Second, the proximity beacon has a large range (5 to 50 meters), thus not allowing for precise inference on physical closeness between the mother and child. Some women’s qualitative reports did not match their passive sensing data. Beyond technical limitations, another explanation may also be that participants reported information they believed the researchers wanted to hear. More validation work is needed to identify whether this is a self-report issue or a technology issue. Third, we only capture passive data during waking hours, limiting information on activities and behaviors that may happen overnight. Fourth, women varied in their household environments and geographic location, where some women experienced more challenges with mobile data access and electricity. We provide extensive reporting on these challenges in another publication [24]. Finally, our sample of women likely exhibits some selection bias given that women from more restrictive families may not allow the participants to freely participate and interact with the research team. As outlined in Maharjan et al, 2021, where we discuss the feasibility and acceptability of passive sensing technology in this population, we highlighted reasons for declining participation to including mothers relocating outside of the study area, inability to contact mothers after initial screening, families not consenting for study participating, and mothers having too many competing obligations.

## Conclusion

We present novel passive data patterns of young mothers’ daily lives, including activity, infant proximity, and geospatial movement throughout the day. In the rural Nepali context, we find that mothers are rarely apart from their infants, remain close to their homes, and have limited activity. The patterns vary within our sample and are largely driven by available household support, her infant’s age and instrumental care requirements, and a mother’s ability to acquire help. Acquiring passive sensing data has exciting opportunity to inform, enhance, and improve behavioral health interventions by providing social, behavioral, and environmental information within an individual’s larger social context that does not require self-report. The mixed-methods approach allows for triangulation of digital data with person-centered accounts of patterns, allowing for a richer interpretation of the findings. Additional research will further refine the technology and approaches to best fit the health needs, culture, and context of the targeted population.

## Supporting information

S1 File COREQ checklist, our qualitative codebook, and the daily diary template. (PDF)

S1 Data (CSV)

## Abbreviations

ANM: Auxiliary nursing midwives
COREQ: Consolidated Criteria for Reporting Qualitative Studies
EBM: Electronic Behavior Monitoring
GPS: Global Positioning System
HIC: High income countries
LMIC: Low- and middle-income countries
StandStrong: Sensing Technologies for Maternal Depression Treatment in Low Resource Settings
