# Supplementary material for: “Now, I have my baby so I don’t go anywhere”: A mixed method approach to the ‘everyday’ and young motherhood integrating qualitative interviews and passive digital data from mobile devices
Source: PLoS One. 2022 Jul 8;17(7):e0269443. doi: 10.1371/journal.pone.0269443 (PMC9269952; doi:10.1371/journal.pone.0269443)
Supplement: S1 File — (PDF) [file pone.0269443.s001.pdf]

Consolidated criteria for reporting qualitative studies (COREQ): 32-item checklist

Developed from:
Tong A, Sainsbury P, Craig J. Consolidated criteria for reporting qualitative research (COREQ): a 32-item checklist for interviews and focus groups. *International Journal for Quality in Health Care*. 2007. Volume 19, Number 6: pp. 349 – 357

**No. Item**	**Guide questions/description**		**Section/Page Number**
**Domain 1: Research team and reflexivity**			
Personal Characteristics			
1. Inter viewer/facilitator	Which author/s conducted the interview or focus group?	N/A	N/A
2. Credentials	What were the researcher's credentials? E.g. PhD, MD	MA, MPH, MSc, MD, PhD,	N/A
3. Occupation	What was their occupation at the time of the study?	Researchers, Associate Professor, Assistant Professors, Interns	N/A
4. Gender	Was the researcher male or female?	Both	N/A
5. Experience and training	What experience or training did the researcher have?	AH, BK, and AvH have PhD level training, and have supervised several PhD and Masters level students in conducting both quantitative and qualitative research. SM has MA in clinical psychology and was the project coordinator. AP is a research associate with MSc in public health. AT has MPH level training. PB has MS and is an IT professional.	N/A
Relationship with participants			
6. Relationship established	Was a relationship established prior to study commencement?	The study team has worked in the community for several years.	Methods//Setting (Page 3)

		Collaboration with the local health system and administrative governance was established prior to study commencment	
7. Participant knowledge of the interviewer	What did the participants know about the researcher? e.g. personal goals, reasons for doing the research	Participants were informed that the researchers were interested in testing the sensing technology to aid the psychosocial intervention.	Data collection (Page 5)
8. Interviewer characteristics	What characteristics were reported about the inter viewer/facilitator? e.g. Bias, assumptions, reasons and interests in the research topic	A brief introduction about the study, organization and interviewer's names and positions provided to the participants before starting the interview. Interviewer-related biases were not identified.	Qualitative Data (Page 5-6)
**Domain 2: study design**			
Theoretical framework			
9. Methodological orientation and Theory	What methodological orientation was stated to underpin the study?	Thematic analysis	Qualitative Data Analysis (Page 6-7)
Participant selection			
10. Sampling	How were participants selected? e.g. purposive, convenience, consecutive, snowball	Convenience sampling	Study Population and Sampling (Page 5)
11. Method of approach	How were participants approached? e.g. face-to-face, telephone, mail, email	Face-to-face interviews	Study Population and Sampling (Page 5)
12. Sample size	How many participants were in the study?	22	Study Population and Sampling (Page 5)
13. Non-participation	How many people refused to participate or dropped out? Reasons?	Not described	Not applicable
Setting			

14. Setting of data collection	Where was the data collected? e.g. home, clinic, workplace	All interviews were conducted at home with initial screening at the health post.	Data collection (Page 5)
15. Presence of non-participants	Was anyone else present besides the participants and researchers?	No	Not applicable
16. Description of sample	What are the important characteristics of the sample? e.g. demographic data, date	Adolescent and young women (15-25 years) with an infant less than one year old.	Study Population and Sampling (Page 5)
Data collection			
17. Interview guide	Were questions, prompts, guides provided by the authors? Was it pilot tested?	Interview guide was prepared and it was rigorously discussed among the authors. Probes were used to facilitate discussions.	Qualitative Data (Page 5-6)
18. Repeat interviews	Were repeat inter views carried out? If yes, how many?	Yes, 3 interview elicitations were conducted for each mother	Qualitative Data (Page 5-6)
19. Audio/visual recording	Did the research use audio or visual recording to collect the data?	Interviews were audio recorded.	Qualitative Data (Page 5-6)
20. Field notes	Were field notes made during and/or after the inter view or focus group?	Field notes after the interview were systematically collected within a template, transcribed and translated and included in the dataset for the analysis.	Qualitative Data (Page 5-6)
21. Duration	What was the duration of the inter views or focus group?	Interviews with women lasted for about 20-45 minutes.	Qualitative Data (Page 5-6)
22. Data saturation	Was data saturation discussed?	No	Not applicable
23. Transcripts returned	Were transcripts returned to participants for comment and/or correction?	No	Not applicable
**Domain 3: analysis and findings**			
Data analysis			

24. Number of data coders	How many data coders coded the data?	Three authors (AH, AP, DLM) coded the data.	Qualitative Data Analysis (Page 6-7)
25. Description of the coding tree	Did authors provide a description of the coding tree?	Yes, the authors generated a coding system and codebook and assessed inter-rater reliability of coding until a kappa greater than .7 was achieved.	Qualitative Data Analysis (Page 6-7)
26. Derivation of themes	Were themes identified in advance or derived from the data?	Themes were derived from the data both inductively and deductively.	Qualitative Data Analysis (Page 6-7)
27. Software	What software, if applicable, was used to manage the data?	NVivo 12.0	Qualitative Data Analysis (Page 6-7)
28. Participant checking	Did participants provide feedback on the findings?	No	Not applicable
Reporting			
29. Quotations presented	Were participant quotations presented to illustrate the themes/findings? Was each quotation identified? e.g. participant number	Yes, quotations were presented to illustrate the themes/findings, and each quotation was identified with an anonymous participant code.	Activity (Starting on page 8)<br><br>Infant Proximity (Starting on page 11)<br><br>Geospatial Movement (Starting on page 14)
30. Data and findings consistent	Was there consistency between the data presented and the findings?	Yes, there was consistency between the data presented and the findings.	
31. Clarity of major themes	Were major themes clearly presented in the findings?	Yes, major themes were clearly presented in the Results section using specific sections regarding each theme.	Activity (Starting on page 8)<br><br>Infant Proximity (Starting on page 11)<br><br>Geospatial Movement

			(Starting on page 14)
32. Clarity of minor themes	Is there a description of diverse cases or discussion of minor themes?	Yes, minor themes were clearly presented in the Results section using specific sections regarding each theme.	Activity (Starting on page 8)<br><br>Infant Proximity (Starting on page 11)<br><br>Geospatial Movement (Starting on page 14)

Themes	Code	Definition	Exclusion	Examples
**Field Notes**	Field Notes	Apply to the entire fieldnote section at the beginning of the transcript. this will then we double coded with any other codes that apply.		
**Support/Care**	Childcare support	Anyone helping with childcare	Mother caring for the baby should be coded under instrumental care	Other family members providing emotional care to the child
	Support in household chores	Anyone helping with household chores	Anything to do with childcare, e.g. washing child's clothes, goes under childcare support	
	General help	Help outside of household chores, e.g. running errands	Not childcare or household chores support	
	Emotional support	Mother getting emotional support	Mother giving emotional support to the baby should be coded under emotional care	
	Trust	Mother trusting or NOT trusting other family members to take care of the baby		Mother says she trusts neighbors over father-in-law
	Informational support on childcare	Mothers learning about childcare from a family member or friends		
**Interaction**	Positive social interaction	Any mention of a positive conversation, discussion with another human being		
	Negative social interaction	Any mention of a negative conversation, discussion with another human being		
	Common/frequent social interactions	Someone mother talks to usually or reaches out to when she wants to talk		E.g., I usually go to my neighbor to talk about my day

	Non-human interaction	YouTube, videos, music, etc.		
	No social interaction	Mother not interested in talking to anyone; silence		
Geospatial Movement	Transportation	Riding a motorcycle, riding a bus/micro, bicycle, etc.	Mentioning moving inside the home	
	Isolation	Mentioning being alone, not joining events or seeking out talking with others		
	Location- outside	Any indication of spending time in a location outside the home (neighbor's house, temple, market, etc.), and participating in social events (weddings, religious outings/going to temple, festivals, visiting family, etc.)	Only code if specifically referring to her own factual movements, not hypothetical or general.	
	Location- home	Indication of spending time inside her own home (not her maternal home).		
**Child proximity**	Instrumental care	Any specific mention of the mother providing tangible care to the child (washing, feeding, changing clothes, putting down to sleep, etc). double code if vague (look after/care for, etc)	Any other members providing childcare support to the mother should be coded under childcare support. Exclude moments when the mother is referring to parenting values.	
	Emotional care	Any mention of the mother providing or describing emotional or developmental care to the child (playing, soothing when crying, speaking, etc) double code if vague (look after/care for, etc)	Any emotional support that the mother gets from others should be coded under emotional support	

	Negative interaction/punishing child	Negative verbal or physical interaction with any child		
	Positive mother-child interaction	Positive verbal or physical interaction with any child, including massage.	Mothers' interaction with other people should be coded under positive social interactions	
	Breastfeeding	Any mention of breastfeeding (starting, stopping, tasks, difficulties, bonding, etc)	instrumental care?	
**Activity**	Household chores	Mentions of engaging in household work such cooking, cleaning, making tea, cleaning dishes, etc.	Other people helping mother with the household chores should go under support in household chores	
	Movement	Mentions of engaging in activities such as walking, running, dancing, riding a motorcycle, riding a bus/micro, bicycle, etc.		
	Waking/sleeping	Any mention of the mother waking up, going to bed, or napping		
	Food mealtimes	Any mention of food consumption, mealtimes, types of food, etc.	Exclude mentions of breastfeeding	
**Mental and physical health**	Positive emotions	Mention of positive affect/emotions (pride, joy, happiness, enjoyment, etc). Frederickson (2009) identifies the ten most common positive emotions as joy, gratitude, serenity, interest, hope, pride, amusement, inspiration, awe, and love.		
	Negative emotions	Mention of negative emotions/emotional states (fear, stress, sadness, depression, stress, worry, tension)		

	Violence	Mention of any emotional, psychological, or physical violence directed towards the mother		
	Fatigue	Any mention of feeling tired or fatigued		
	Physical health	Any mention of physical health and well-being		
	Identity	Mention of wanting to become a mother, what it was like now that she is a mother, or mentions of being a daughter-in-law, etc.		
	Coping/help seeking	Indicating coping strategies (dancing, listening to music, etc), seeking/asking for help at a health center, through friends, family etc, or purposefully NOT seeking help	If she is getting help/support, code under support/care meta code (emotional support, childcare support).	Compliance is the 'right thing to do'; buhari agency is limited in hierarchical family structures
	Parenting values	Mention of approaches to parenting (positive reinforcement, permissive, authoritative) or hopes and values to attain through parenting (shaping a 'good' child, etc).		
	Alcohol	Any mention of anyone drinking alcohol at any time		
	Cultural practices	Returning to natal home, oil massage, isolation/confinement, not celebrating particular holidays, etc. (examples can include mentions of sutkeri (mom that just gave birth, up until 6 months)		Sutkeri (mom that just gave birth, up until 6 months) (will not celebrate major holidays for 10 days)
**Socio-demographics**	Work	Mention of formal or informal work (excluding housework) done by the mother or other family members		

	Poverty	References to money, poverty, income, etc.		
	Child gender	Mention of the child's gender		

Participant Code: **Day in a Life** Date:

Morning						
**Time**	**Activity**	**Location**	**Child's activity/location/with whom**	**Who else is with you?**	**Feeling/Mood**	**Notes**
4:00 AM					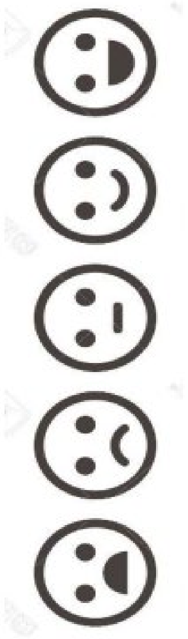	**Wake up Time:**
5:00 AM						
6:00 AM						
7:00 AM						
8:00 AM						
9:00 AM						
10:00 AM						
11:00 AM						

Afternoon						
**Time**	**Activity**	**Location**	**Child's activity/location/with whom**	**Who else is with you?**	**Feeling/Mood**	
12:00 PM						
1:00 PM						

Participant Code: **Day in a Life** Date:

2:00 PM					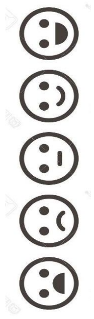	
3:00 PM						
4:00 PM						
**Evening**						
**Time**	**Activity**	**Location**	**Child's activity/location/with whom**	**Who else is with you?**	**Feeling/Mood**	**What time did you go to bed?**
5:00 PM					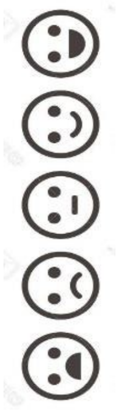	
6:00 PM						
7:00 PM						
8:00 PM						Sleep time:

Did you sleep well throughout the night?

Were there any interruptions to sleep? If yes, what caused them? For eg: breastfeeding or changing clothes of child, etc.
